# Oral and pharyngeal cancer in South Asians and non-South Asians in relation to socioeconomic deprivation in South East England

**DOI:** 10.1038/sj.bjc.6604191

**Published:** 2008-01-22

**Authors:** D R Moles, S Fedele, P M Speight, S R Porter, I dos Santos Silva

**Affiliations:** 1 Health Services Research, UCL Eastman Dental Institute, 256 Grays Inn Road, London WC1X 8LD, UK; 2 Department of Oral Medicine, UCL Eastman Dental Institute, 256 Grays Inn Road, London WC1X 8LD, UK; 3 Department of Oral and Maxillofacial Pathology, The School of Clinical Dentistry, The University of Sheffield, Claremont Crescent, Sheffield S10 2TA, UK; 4 Cancer Research UK Epidemiology and Genetic Group, London School of Hygiene and Tropical Medicine, Keppel Street, London WC1E 7HT, UK

**Keywords:** oral cancer, incidence, ethnicity, relative deprivation, England

## Abstract

From UK Thames Cancer Registry data, after controlling for socioeconomic deprivation of area of residence, South Asian males showed a higher relative risk of oral (1.36; 95% CI: 1.11, 1.67), but not of pharyngeal cancer than non-South Asian males, whereas South Asian females had much higher risks of these cancers (3.67; 95% CI: 2.97, 4.53 and 2.06; 95% CI: 1.44, 2.93), respectively, than non-South Asians.

South Asians with origins in India, Pakistan and Bangladesh constitute the largest minority ethnic group in Britain, representing 4% of the 2001 English population (Office for National Statistics, 2003). At lower risk of most cancers, they have increased risks of oral and pharyngeal cancers relative to the general British population (Winter *et al*, 1999). Variations in the risk of cancers are strongly related to the prevalence of chewing of tobacco, areca nut and betel quid in developing countries (Lay *et al*, 1982; Ikeda *et al*, 1995; Gupta and Nadakumar, 2000) and of tobacco smoking and high alcohol consumption in developed countries (Blot *et al*, 1988; La Vecchia *et al*, 1997; Hindle *et al*, 2000a, 2000b). Ethnic differences in the incidence of these cancers are little studied in Britain, mainly because of the lack of reliable ethnicity data in cancer registries. We investigated differences in the incidence of oral and pharyngeal cancers between South Asians and non-South Asians in England, taking into account their socioeconomic status and population-based data on ethnic differences in tobacco use and alcohol intake.

## MATERIALS AND METHODS

With ethical approval from the London School of Hygiene and Tropical Medicine ethics committee, data for all oral and pharyngeal incident cancers diagnosed in the south east of England in 1985–1995 were obtained from the Thames Cancer Registry (TCR). As ethnicity data were incomplete in TCR files, a computer algorithm, SANGRA (South Asian Names and Group Recognition Algorithm) of high sensitivity and specificity (Nanchahal *et al*, 2001), was used to identify persons of South Asian origin on the basis of their names. Socioeconomic status at diagnosis was ascertained by the Carstairs index (Carstairs and Morris, 1989). Each of the 1999 census wards in England and Wales was assigned a deprivation score and the resulting distribution categorised into five groups (1=least deprived to 5=most deprived). Postcode of usual residence at diagnosis was used to allocate each subject to a ward and, hence, to a deprivation category.

Analyses were conducted separately for oral (ICD-10: C01–C06) and pharyngeal (ICD-10: C09, C10, C12–C14) cancers. The relative importance of tobacco products and alcohol intake has been found to differ for these cancers (Franceschi *et al*, 1999; Hindle *et al*, 2000a, 2000b). Age-standardised incidence rates (per 100 000 person-years) were calculated by the direct method, in 5-year age bands, to the World Standard Population. Population denominators by ethnicity were obtained from the 1991 census; people who defined themselves as ‘Indian’, ‘Pakistani’ and ‘Bangladeshi’ were regarded as South Asians. Poisson regression models were fitted to estimate age-adjusted and age-deprivation-adjusted incidence rate ratios (Breslow and Day, 1993). Models were compared that included and excluded the deprivation quintiles, respectively; these were modelled both as a series of dummy variables and as a linear trend (nested models), with their relative fit compared using likelihood ratio tests.

In the late 1990s, the government commissioned the first nationally representative survey into health-related behaviours among minority ethnic groups in England. More than 1000 adults in each of the main three South Asian groups (Indian, Pakistani and Bangladeshi) were interviewed (Department of Health, 2001). Data on tobacco use and alcohol intake, by ethnicity, were extracted from the Survey's report (http://www.archive.official-documents.co.uk/document/doh/survey99/hse99.htm).

## RESULTS

The TCR held 6658 registrations of incident oral and pharyngeal cancers in 1985–1995, with 6355 (95.4%) having data on age, gender and postcode situated within its catchment area. SANGRA identified 282 (4.4%) registrations as being of South Asian origin. South Asians had higher age-adjusted incidence rates of these cancers than non-South Asians (Table 1), particularly among females. Among non-South Asians, males had over twice the risk of females. In contrast, South Asian males had a 15% lower risk of oral but a 63% higher risk of pharyngeal cancer than South Asian females.

The incidence of both oral and pharyngeal cancers increased with increasing socioeconomic deprivation of area of residence among non-South Asian males, the most deprived having over twice the risks of those in the least deprived quintile (Table 2). Among non-South Asian females, there was also a positive trend in pharyngeal cancer risk with deprivation, although less marked than in males, but no clear trend in oral cancer risk. Neither cancer was associated with deprivation among South Asians. After adjustment for both age and deprivation of area of residence (Table 3), there was only a small ethnic difference in oral cancer incidence in males, and none in pharyngeal cancer. In contrast, South Asian females had substantially greater risks of these cancers than non-South Asian females (Table 3).

These ethnic differences do not parallel the ethnic variations in alcohol intake reported by the 1999 Health Survey for England. Mean weekly consumption of alcohol units by South Asians (range: 0.0–8.6) and proportion of heavy drinkers (range: 0–14%) were substantially lower than among the general population (7.2–17.5 and 16–30%, respectively). After adjustment for under-reporting as ascertained by saliva cotinine levels, current use of any form of tobacco product was substantially higher among Bangladeshi males (relative risk (RR)=1.52) and females (RR=1.75), mainly due to tobacco chewing, than in the general population (RR=1.00). Prevalence in Pakistani males (RR=0.97) was similar to that in the general population, but much lower in Indian (RR=0.38) and Pakistani (RR=0.31) females. Socioeconomic status was negatively associated with tobacco consumption, but positively associated with alcohol intake in the general population; there were no clear trends among South Asians.

## DISCUSSION

The oral and pharyngeal cancer rates observed here are of similar magnitude to those previously estimated among English South Asians (Winter *et al*, 1999), but much lower than in the Indian subcontinent (Ferlay *et al*, 2004). Others have also found that these cancer risks in migrants are intermediate between those of the host and the countries of origin (Grulich *et al*, 1995; Swerdlow *et al*, 1995; Jain *et al*, 2005), probably reflecting changes in behaviour.

The positive trend in oral and pharyngeal cancer risks with socioeconomic status in non-South Asians is consistent with the socioeconomic differences in the consumption of tobacco, but not alcohol, reported by the 1999 Health Survey. Similarly, the ethnic differences in risk in females in our study do not parallel differences in their alcohol consumption but are, to a certain extent, consistent with their higher prevalence of tobacco chewing, particularly among Bangladeshi women. This would accord with tobacco chewing being associated with higher risks of oral than pharyngeal cancers (Dikshit and Kanhere, 2000), the former effect being stronger in females (Balaram *et al*, 2002). A substantial limitation of the Health Survey is its not covering the use of betel alone, a well-established carcinogen (Chang *et al*, 2005); its use is common in some South Asian (and other) minority ethnic groups (Bedi and Gilthorpe, 1995).

Our study, like similar investigations, was limited by misclassification in assigning of ethnicity on the basis of names and by its inability to distinguish subethnic groups within the South Asian population, despite evidence that risk behaviours vary greatly according to religion and region of origin. Nevertheless, the high risks of oral and pharyngeal cancers presumably reflect their higher consumption of tobacco products and betel quid alone.

## Figures and Tables

**Table 1:** Oral and pharyngeal cancer incidence by ethnicity, gender and relative deprivation, South East England, 1985–1995

		**Non-South Asians**				**South Asians**			
**Anatomical site**	**Deprivation quintile**	**Males**		**Females**		**Males**		**Females**	
**(ICD-10)**	**(Carstairs)**	***N***	**Rate (95% CI)^a^**	***N***	**Rate (95% CI)^a^**	***N***	**Rate (95% CI)^a^**	***N***	**Rate (95% CI)^a^**
Oral cavity (C01–C06)	1 (least deprived)	246	1.44 (1.25, 1.63)	248	1.18 (1.01, 1.34)	6	5.68 (0.54, 10.85)	8	8.49 (2.10, 14.89)
	2	311	1.77 (1.56, 1.97)	247	0.98 (0.84, 1.12)	8	3.56 (0.83, 6.29)	5	3.29 (0.29, 6.29)
	3	380	2.06 (1.84, 2.27)	299	1.21 (1.05, 1.36)	8	2.74 (0.66, 4.82)	8	3.46 (0.94, 5.97)
	4	499	2.38 (2.16, 2.60)	361	1.25 (1.10, 1.40)	25	4.70 (2.76, 6.64)	27	4.70 (2.83, 6.56)
	5 (most deprived)	801	3.46 (3.21, 3.70)	412	1.23 (1.10, 1.37)	54	3.80 (2.71, 4.89)	50	4.70 (3.34, 6.07)
	All quintiles	2237	2.31 (2.21, 2.40)	1567	1.17 (1.11, 1.24)	101	3.97 (3.14, 4.80)	98	4.65 (3.68, 5.62)
									
Pharynx (C09, C10, C12–C14)	1 (least deprived)	157	0.93 (0.78, 1.07)	104	0.47 (0.37, 0.57)	1	0.45 (0.00, 1.32)	3	3.40 (0.00, 7.34)
	2	198	1.07 (0.91, 1.22)	111	0.47 (0.37, 0.57)	5	3.64 (0.31, 6.98)	2	0.92 (0.00, 2.20)
	3	237	1.21 (1.05, 1.37)	133	0.51 (0.41, 0.61)	3	2.02 (0.00, 4.30)	2	0.66 (0.00, 1.57)
	4	330	1.60 (1.41, 1.78)	197	0.76 (0.64, 0.87)	11	2.45 (0.95, 3.95)	8	1.49 (0.41, 2.56)
	5 (most deprived)	547	2.39 (2.18, 2.60)	255	0.86 (0.74, 0.97)	30	2.16 (1.33, 3.00)	18	1.36 (0.69, 2.03)
	All quintiles	1469	1.51 (1.43, 1.59)	800	0.63 (0.58, 0.68)	50	2.24 (1.57, 2.90)	33	1.37 (0.87, 1.86)

CI=confidence interval.

a Incidence rate (with 95% confidence interval) per 100 000 person-years, age standardised in 5-year age bands to the World Standard Population.

**Table 2:** Risk of oral and pharyngeal cancers by relative deprivation of area of residence in South Asians and non-South Asians resident in South East England, 1985–1995

		**Males**		**Females**	
**Anatomical site**	**Deprivation quintile**	**Non-South Asian**	**South Asian**	**Non-South Asian**	**South Asian**
**(ICD-10)**	**(Carstairs)**	**IRR (95% CI)**	**IRR (95% CI)**	**IRR (95% CI)**	**IRR (95% CI)**
Oral cavity (C01–C06)	1 (least deprived; reference)	1	1	1	1
	2	1.18 (1.00, 1.40)	0.84 (0.29, 2.43)	0.89 (0.75, 1.07)	0.38 (0.12, 1.15)
	3	1.35 (1.15, 1.58)	0.58 (0.20, 1.67)	0.98 (0.83, 1.16)	0.41 (0.15, 1.10)
	4	1.60 (1.37, 1.86)	0.85 (0.35, 2.07)	1.04 (0.89, 1.23)	0.63 (0.29, 1.39)
	5 (most deprived)	2.20 (1.91, 2.54)	0.77 (0.33, 1.79)	1.05 (0.90, 1.23)	0.54 (0.25, 1.13)
					
	Trend per quintile	1.22 (1.18, 1.26)	0.98 (0.83, 1.15)	1.03 (0.99, 1.07)	0.96 (0.82, 1.14)
	*P*-value from likelihood ratio test	0.105	0.736	0.436	0.234
					
Pharynx (C09, C10, C12–C14)	1 (least deprived; reference)	1	1	1	1
	2	1.18 (0.96, 1.46)	3.02 (0.35, 25.84)	0.96 (0.74, 1.26)	0.40 (0.07, 2.42)
	3	1.32 (1.08, 1.62)	1.24 (0.13, 11.86)	1.05 (0.81, 1.36)	0.28 (0.05, 1.65)
	4	1.67 (1.38, 2.02)	2.06 (0.27, 15.96)	1.38 (1.08, 1.74)	0.50 (0.13, 1.90)
	5 (most deprived)	2.38 (1.99, 2.84)	2.30 (0.31, 16.85)	1.57 (1.25, 1.97)	0.51 (0.15, 1.74)
	Trend per quintile	1.25 (1.20, 1.30)	1.08 (0.83, 1.40)	1.15 (1.09, 1.20)	0.97 (0.73, 1.29)
	*P*-value from likelihood ratio test	0.093	0.541	0.310	0.553

95% CI=95% confidence interval; IRR=age- and deprivation-adjusted incidence rate ratio.

**Table 3:** Risk of developing oral and pharyngeal cancer in South Asians relative to non-South Asians (reference), South East England, 1985–1995, adjusted for age and deprivation

**Anatomical site**	**Males**		**Females**	
**(ICD-10)**	**IRR (95% CI)**	***P*-value**	**IRR (95% CI)**	***P*-value**
Oral cavity (C01–C06)	1.36 (1.11, 1.67)	0.003	3.67 (2.97, 4.53)	<0.001
Pharynx (C09, C10, C12–C14)	1.00 (0.75, 1.34)	0.978	2.06 (1.44, 2.93)	<0.001
Oral and pharynx combined	1.22 (1.03, 1.44)	0.019	3.07 (2.56, 3.68)	<0.001

95% CI=95% confidence interval; IRR=age- and deprivation adjusted incidence rate ratio.
